# Supplementary material for: Overexpression of PpSnRK1α in Tomato Increased Autophagy Activity under Low Nutrient Stress
Source: Int J Mol Sci. 2022 May 13;23(10):5464. doi: 10.3390/ijms23105464 (PMC9141306; doi:10.3390/ijms23105464)
Supplement: Supplementary file 1 [file ijms-23-05464-s001.zip › ijms-1716822-supplementary.pdf]

Table S1: Primers used in this study

Gene	Forward sequence (5’-3’)	Reverse sequence (5’-3’)
PpSlSnRK1α	GAGGTGGTTAAGATGGGAT	TCAGCTCCAAGATAGCCA
Actin SlEF1α	TGGAAACGGATATGCCCCTG	TGGGCTTGGTGGGAATCATC
SlSnRK2.1	CAGTTTGAGGAGCCAGATCA	GTCATCGTCAATGTCCAAGC
SlSnRK2.2	AGCTGATGGATGTTGAGCAC	GCAGGATCTTTCCCTTCAGA
SlSnRK2.3	GAAGAACCCGATCAACCAAT	CAGGCTCTGAATCCAAGTCA
SlSnRK2.4	GATGATGAAATTGACACAAGTGG	CATGGCTTGAACGTGTATGA
SlSnRK2.5	CAATGGCATACCACAATCCT	AGGGCTTCGCTTTGTAGTTG
SlSnRK2.6	GGAACTGATGGAAGGAGGAA	CCTGCAGAGGAACTCTAGCC
SlSnRK2.7	CAATGCAGGAAGGTTCAGTG	GAGCTGCACTTCCATCAAGA
SlATG1a	GATCGTTACGGCTCGGCTTA	CTTCAAGGTGGAGCGGTCAT
SlATG2	AGCAGCGGATGCTAACCTTT	GACGGGACTCAGACAGCAAA
SlATG3	TCACAGCTTCACGGAGCATT	ATTCGCTGACGCTGAGAACA
SlATG4	TGGGGTTGCATGCTTAGGAG	CAAGCGGCTGATTGTGCATT
SlATG5	CCTGAACGGCCTTGGAATCT	CCATAATGGAGCGCCAGAGT
SlATG6	GCTACACCGGATAAGGGTCG	GCATCCCGGAGCGATAAGAA
SlATG7	AGTCGGTGGCTTCCTAGTCT	GGGTACGGTTAGCCGTTGAA
SlATG8c	TGCGGAAGAGGATCAAGCTC	TTCCAAGCTCAACGAACCCA
SlATG9	TCAACAGCAGTGCCGTACAT	TGTTATTGCAGCCCGGCTTA
SlATG10	TATACTGCGCAGGAACTGGC	GTACCATGGCCGATTCAGGT
SlATG12	TGGCGATGCTCCGATTCTTA	CTCCCTATGGAGTTGACGGC
SlATG13	CTGCCCATTCACCACAAAGC	CAAACGAGTCTGCCCAGGAT
SlATG18b	GTCATCGTCAATGTCCAAGC	CACGTGTTCAGGTGTTGCTG
